# Impact of Online-Delivered eHealth Literacy Intervention on eHealth Literacy and Health Behavior Outcomes among Female College Students during COVID-19

**DOI:** 10.3390/ijerph20032044

**Published:** 2023-01-22

**Authors:** Miyoung Roh, Yoonkyung Won

**Affiliations:** 1 College of General Education, Kookmin University, 77, Jeongneung-ro, Seongbuk-gu, Seoul 02707, Republic of Korea; 2 Department of Physical Education, Yonsei University, Seoul 03722, Republic of Korea

**Keywords:** online eHealth literacy intervention, eHealth literacy, exercise self-schemata, health behavior, female college students, COVID-19

## Abstract

This study examined the effects of a novel online-delivered eHealth literacy intervention to improve eHealth literacy and positive health behaviors among female college students during COVID-19. Female college students taking a physical education class were allocated to either an online-based eHealth literacy intervention group (*n* = 62) or a physical education class (*n* = 58). Weekly two-hour sessions were implemented through Zoom videoconferencing over six weeks. We measured eHealth literacy, exercise self-schemata, and health behavior outcomes (eating, sleep, and exercise) before and after the intervention. A two-way repeated measures ANOVA was conducted to examine within- and between-group differences in all outcomes. The ANOVA (2 × 2) for the interaction effect of group and time showed a statistical significance in eHealth literacy and cognitive-emotional exercise self-schemata. There was a marginally significant interaction effect for exercise but none for eating and sleep. This was the first trial to examine the impact of the online eHealth literacy intervention on eHealth literacy and health behavior outcomes for college students during COVID-19. Preliminary findings indicated that the intervention showed promising effectiveness for improving eHealth literacy and promoting health behaviors among female college students.

## 1. Introduction

College students’ health behavior in the transition to adulthood is essential for a healthy lifestyle throughout adulthood [1,2]. However, studies have shown that they are exposed to various health risks [3,4,5]. Newly admitted students are more vulnerable while adapting to a new environment, such as a university [4]. Furthermore, those joining after COVID-19 experienced additional stress related to classes changing from in-person to online [6]. Dziewior [7] reported that all activities in universities were changed to online during COVID-19; consequently, physical activity decreased, and sedentary life became more prominent. Therefore, it is necessary to focus more on their healthcare.

COVID-19 accelerated the transition to a digital society; eHealth literacy (eHL) is the ability to accurately search for and utilize health-related information using technology such as the Internet [8] and is emerging as important for maintaining a healthy life. Most college students (99%) use the Internet as an essential tool to obtain health-related information [9]; eHL is a significant factor in maintaining health and preventing disease [10]. Hsu et al. [11] reported that college students with higher eHL promoted positive health behaviors, such as healthy eating, exercise, and sleep. Conversely, lacking this skill can adversely affect disease prevention and healthcare [12].

Many studies have indicated that eHealth literacy among college students is insufficient [10,13,14]. Stellefson et al. [10] noted that, despite their perceived ability to use the Internet, they lacked eHL. Moreover, eHL differed between male and female students, with the latter obtaining more health-related information from the Internet than male students [15] but having a lower ability to utilize and practice it [14]. Therefore, college students with low eHealth literacy are likely to search for and exploit misinformation, which can negatively impact their health outcomes. Thus, tailored intervention research is needed to improve the eHL and health of new college students, who are more vulnerable to healthcare.

Several studies have examined interventions that promote positive health behaviors, such as physical activity [16,17,18,19,20], healthy diets [21,22,23], and mental health [21,24]. However, few have investigated interventions focusing on promoting eHL and health behavior outcomes among college students through online methods using the Internet. Although many studies reported that eHL positively affects health behavior outcomes [11,25,26], there were gender differences [27]. Moreover, a negative association between eHL and balanced diet has been reported regarding female students [28]. Tsukahara [29] indicated that further research is needed as it is unclear whether an intervention focused on eHL could help improve students’ health behaviors. Therefore, this study developed and applied an online-delivered eHL intervention among newly admitted female college students during COVID-19 and examined its impact on improving eHealth literacy and positive health behaviors.

Previous studies have focused on investigating the association between eHL and behavioral health outcomes [11,25,27,30]. Recently, Roh [31] found that eHL is a crucial factor influencing the improvement of exercise self-schemata, a cognitive health behavior. Self-schemata in exercise behavior are an important cognitive structure of knowledge about exercise and a domain of self-perception that influences how to perceive exercise through experience and health knowledge and the motivation to participate in exercise [32]. Previous studies have shown that college students with exercise self-schemata participated in exercise more frequently [33,34] and had a higher intention to continue to exercise than those without [35,36]. In other words, exercise self-schemata positively affected the practical aspects of participating in exercise and future exercise behavior.

However, despite the health benefits of positive health behaviors, the exercise practice rate in South Korea is 20.8%, which is about half of the number of American college students [37]. Unlike the United States, female college students in South Korea are much less likely to participate in exercise (13.6%) compared to male students (34.2%) [38] and more likely to have low exercise self-schemata [39]. Moreover, a survey by the Korea Institute of Health and Social Affairs [40] reported that only 29.1% of adults in South Korea had an appropriate health literacy level, and many searched for health information mainly on the Internet; hence, a large number of adults had difficulty understanding, evaluating, and utilizing health-related information obtained through the Internet. To the best of our knowledge, no studies have examined the eHL intervention among college students. Therefore, this study aimed to develop and apply a novel, online-delivered eHL intervention to improve the eHL of female college students during COVID-19 and identify its effect. The study also examined whether eHL intervention would be effective in improving health behavior outcomes, which are closely related to eHL.

## 2. Materials and Methods

### 2.1. Participants

The study participants were female college students enrolled in a physical education (PE) class at a private woman’s college in Gyeonggi-do, Republic of Korea. In this study, we recruited female students who were more vulnerable than male students in eHealth literacy [27] and health behaviors [38,39]. To determine the sample size, calculation was performed using the G*Power software (Statistical power analyses, G*Power 3.1, Germany). The minimum sample size was defined as 84 participants: power (1-β) = 0.95, significance level (α) = 0.05, effect size = 0.20. To ensure a sufficient sample size, four PE classes (*n* = 124; 31 students per class) were purposely included and randomly assigned by coin toss to either the eHL intervention group (two intervention classes) or the regular PE group (two control classes). All participants volunteered to participate in this study. The four participants in the PE group who did not complete the post-test were excluded from the analysis. The final analysis included eHL group (*n* = 62; age = 19.4 ± 0.5 years) and PE group (*n* = 58; age = 19.5 ± 0.6 years).

### 2.2. Procedure and Structure of Intervention

The experimental group participated in a six-week online eHL intervention program during PE classes. The intervention combined physical education (vinyasa yoga) and a specially designed eHL education program (two hours total) to improve eHL and induce health behavior change. The control group participated in the existing vinyasa yoga PE class curriculum (vinyasa yoga participation and education program) provided by university PE instructors, with research procedures and period matched with those of the experimental group. The control group, however, did not receive any information and contents of the eHL education program. All classes were conducted using Zoom software (Zoom Video Communications, Inc., San Jose, CA, USA).

**Figure 1:**
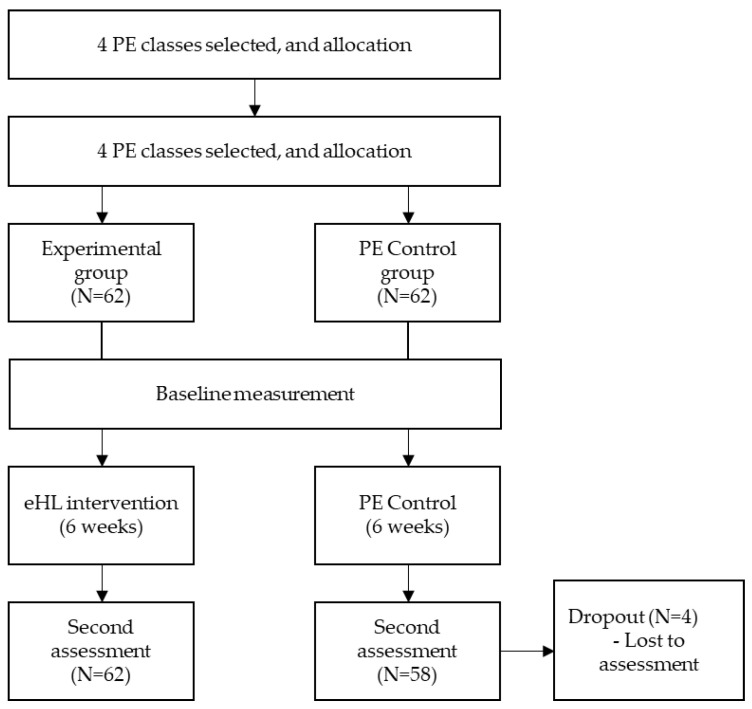
Participant enrollment and assessment flowchart. PE: Physical Education; eHL: eHealth Literacy.

The eHL intervention comprised weekly two-hour sessions during PE classes over six weeks. The eHL education program contents were based on materials from previous studies on eHL programs [41,42] and evidence-based health literacy education programs among female college students in PE classes [43]. An intervention duration of six weeks or less has been tested and proven to be effective in previous eHL intervention studies [41,42,43]. We developed a six-session eHL education program tailored to the participants of this study through several meetings with relevant experts, and the final version of each session was produced after several careful modifications to the program. The six-session online eHL intervention program comprised two sequential steps: (1) health-related knowledge-building education for acquiring basic information on positive health behaviors (e.g., physical activity, healthy eating, and sleep), (2) eHL experience-building practice for allowing students to find, assess, and utilize reliable health resources online through a practice program focused on promoting eHL. The two sequential steps (health education and practice) are more effective when health education and related practice are combined in the promotion of health behavior [44]. Specifically, we conducted practical training using a credible online health information portal site in South Korea: the Korea National Health Information Portal provided by the Korea Disease Control and Prevention Agency, promoting eHL. The practice was based on a previous study [45] showing that eHL was higher in adolescents familiar with MedlinePlus, a reliable health-related site. Particular session goals and contents of each session of the eHL education program are presented in Table 1. Figure 1 represents participant allocation and a flow diagram that shows the research process before and after the intervention.

Before the study, the purpose and procedure of the experiment were fully explained to the participants. Online surveys were administered through Google Forms before and after the intervention, and all participants received a small gift (e.g., coffee coupon) as a reward for their participation. After the entire experiment was completed, considering research ethics, each group was provided with the other group’s intervention.

### 2.3. Measures

#### 2.3.1. Demographics

To examine participants’ general characteristics, we investigated their age, academic year, major, and time spent on the Internet. Health-related characteristics included items on subjective health status, health interest, and weekly exercise time.

#### 2.3.2. eHL

For the analysis, eHL was measured using the eHealth Literacy Scale (eHEALS) [8] which was translated and modified into Korean [46]. The Korean version of the scale is a reliable tool which validity has been confirmed with a reliability coefficient of 0.88 [46]. The scale comprised 8 items scored on a 5-point Likert scale (1 = strongly disagree to 5 = strongly agree), with higher scores indicating a higher eHL level. The reliability coefficient (Cronbach’s α) of this study was 0.93.

#### 2.3.3. Exercise Self-Schemata (ESS)

Exercise self-schemata were measured using the Exercise Self-Schemata (ESS) questionnaire [47]. The questionnaire comprised 14 items with two subscales: behavioral exercise self-schemata (BESS; 7 items) and cognitive-emotional exercise self-schemata (CEESS; 7 items). Each item was scored on a 5-point Likert scale from 1 (strongly agree) to 5 (strongly disagree), with higher scores indicating higher exercise self-schema. The reliability coefficient (internal consistency) was 0.91 and 0.88 for the behavioral and cognitive-emotional exercise self-schemata, respectively.

#### 2.3.4. Health Behavior

We used the Health Behavior Scale (HBS) [11] to measure college students’ health behavior. This scale has 12 items that contain the following three subscales: eating (4 items), exercise (4 items), and sleep (4 items). Each item was scored on a scale from 1 (strongly agree) to 5 (strongly disagree), with higher scores indicating better health-related habits. The reliability coefficients were eating (0.61), exercise (0.79), and sleep (0.65).

### 2.4. Study Design and Ethics

A controlled, quasi-experimental study recruited female college students enrolled in four PE classes at a women’s university in Gyeonggi-do, Republic of Korea. Two classes were randomly allocated to the eHL intervention (experimental group) or PE class (control group). Both were delivered online using Zoom software. We used a 2 × 2 mixed factorial design with two groups (experimental group; control group) as the between-subjects variable and the measurement (pre-test; post-test) as the within-subjects variable to examine the effect of the intervention on eHL, exercise self-schemata, and health behavior in female college students during COVID-19. We used the online platform Zoom version 5.4, because of its end-to-end encryption, convenience, accessibility and cost-effectiveness, which became commonplace in educational institutions during the pandemic [48]. Considering participants’ privacy and ethics issues, we posted the Zoom link on a secure university website and asked the students not to share their personal information to other students. The protocol of this study was approved by the Institutional Review Board of Sunmoon University (SM-202009-066-2). Informed consent was obtained from all participants involved. The collected data were not used for purposes other than research. Personal information of participants was protected by the Personal Information Protection Act (PIPA).

### 2.5. Statistical Analysis

Descriptive analyses were conducted for the general characteristics and outcome variables. A Chi-square (χ^2^) test and independent t-tests were performed to verify the homogeneity between the experimental and control groups. A total of six separate two-way repeated measures ANOVAs examined the comparison of the main effect between group (experiment group; control group), time (pre-test; post-test) and their interactions on eHL, exercise self-schemata (BESS, CEESS), and health behavior (eating, exercise, sleep). A partial eta squared (η^2^) was calculated to explain effect sizes for main effects and their interactions. We calculated 95% confidence intervals to estimate the range of parameters. All statistical analyses were performed using IBM SPSS Statistics for Windows, version 25.0 (IBM Corp., Armonk, NY, USA).

## 3. Results

### 3.1. Participants and Homogeneity Test

The study participants’ general characteristics and the homogeneity test between the experiment (*n* = 62) and control groups (*n* = 58) are presented in Table 2. The participants’ mean ages were 19.4 ± 0.5 years and 19.5 ± 0.6 years in the experimental and control groups, respectively, and they were all female college students. As a result of the homogeneity test, there were no group differences in the baseline general characteristics, eHL (*t* = −1.26, *p* = 0.211), exercise self-schemata (BESS: *t* = −1.82, *p* = 0.071; CEESS: *t* = −1.76, *p* = 0.080) and health behavior (eating: *t* = −0.22, *p* = 0.825; sleep: *t* = −0.83, *p* = 0.408; exercise: *t* = 0.957, *p* = 0.341), ensuring homogeneity between the two groups.

### 3.2. Changes in eHealth literacy

Table 3 shows the result of the two-way ANOVA with repeated measures to examine the effects of the intervention on eHL between the experimental and control groups. The results revealed that the interaction effect of the group and time (F(1, 118) = 4.765, *p* = 0.031, partial η^2^ = 0.039) showed a statistically significant difference. The main effect for time (F(1, 118) = 8.277, *p* = 0.005, partial η^2^ = 0.066) was statistically significant, and for the group it was not statistically significant (F(1, 118) = 0.064, *p* = 0.800, partial η^2^ = 0.001). The experimental group showed a significant increase in eHL compared to the control group after the intervention.

### 3.3. Changes in Exercise Self-Schemata

A two-way ANOVA with repeated measures was perform to examine the effects of the intervention on behavioral exercise self-schemata and cognitive exercise self-schemata between the experimental and control groups. This is presented in Table 4. For behavioral exercise self-schemata, there was no statistically significant main effect for group (F(1, 118) = 2.634, *p* = 0.107, partial η^2^ = 0.022) and the interaction effect of group and time (F(1, 118) = 1.494, *p* = 0.224, partial η^2^ = 0.013). There was statistical significance for time (F(1, 118) = 6.092, *p* = 0.015, partial η^2^ = 0.049). For cognitive exercise self-schemata, there was a statistically significant difference in the interaction effect between group and time (F(1, 118) = 5.648, *p* = 0.019, partial η^2^ = 0.046). There was no significant main effect for group (F(1, 118) = 0.026, *p* = 0.873, partial η^2^ = 0.000) and time (F(1, 118) = 0.611, *p* = 0.436, partial η^2^ = 0.005). The experimental group showed a significant increase in cognitive exercise self-schemata after the intervention, while the control group showed no significant difference in pre-test and post-test.

### 3.4. Changes in Health Behavior

Table 5 shows the result of a two-way ANOVA with repeated measures to investigate the effects of the intervention on health behavior between the experimental and control groups. The results revealed that the interaction effect of group and time (F(1, 118) = 0.142, *p* = 0.707, partial η^2^ = 0.001 for eating; F(1, 118) = 0.793, *p* = 0.375, partial η^2^ = 0.007 for sleep) did not show a statistically significant difference. There was statistically significant main effect for time (F(1, 118) = 12.923, *p* = 0.000, partial η^2^ = 0.099 for eating) and group (F(1, 118) = 10.793, *p* = 0.001, partial η^2^ = 0.084 for exercise). For exercise, there was marginal significance at *p* < 0.10 [49] for the interaction effect between group and time (F(1, 118) = 3.474, *p* = 0.065, partial η^2^ = 0.029). The results indicated that the experimental group showed a marginally significant increase in exercise than the control group after the intervention.

## 4. Discussion

This study aimed at obtaining new knowledge about a novel, online eHL intervention to improve eHL among female college students during COVID-19. The main finding of this study was that eHL increased significantly after the intervention compared to PE control conditions. Furthermore, cognitive-emotional exercise self-schemata significantly improved after the intervention. However, there were no significant interaction effects for health behavior outcomes such as exercise, eating, and sleep.

To the best of our knowledge, this is the first pilot interventional study targeted at female college students to use online eHL intervention in a college PE course and verify its effects on eHL and closely related health behavior outcomes. The main finding indicated that online eHL intervention was accessible for female college students and effectively improved it, as well as promoted cognitive exercise self-schemata which has been related to eHL. This positive result is consistent with the evidence from a previous study that eHL would be essential in the improvement of exercise self-schemata in female college students [31]. These results suggest that eHL education contents on the cognitive aspects of how exercise is perceived by individuals and informational elements such as its effects may increase the exercise self-schemata of cognitive aspects. Moreover, considering that the participants were Korean female college students who were vulnerable to healthcare, the effects of the intervention are positive, and it is encouraging that it would be an important factor in influencing the improvement of cognitive exercise self-schemata among female college students with low levels of physical activity. The findings can be supported based on previous studies that show that exercise self-schematic [35,50] and eHL [11,25,27] positively impact various positive health outcomes, and they are closely related [31] in cross-sectional studies. However, these findings should be cautiously interpreted, as participants were limited to Korean female college students only.

Given that both students and instructors are having difficulties following the class arrangement due to non-face-to-face conversion during the COVID-19 pandemic [51], the practical purpose of this study was to develop an online-based eHL intervention and apply it to the actual PE class to test its field applicability. The development of online materials in the education field were further expanded after COVID-19, and this study implies that online-based eHL intervention could be of great significance in health-related classes and their accessibility to college students. The results of this study have important practical implications in health-related classes on college campuses. Additionally, they can be easily provided to other universities across the country to improve college students’ eHealth literacy. Thus, based on the online eHL intervention developed in this study, additional research is needed to generalize the effectiveness by expanding and applying it to various health-vulnerable groups; not only to college-aged populations but also to the elderly and people with disability.

This study also examined whether eHL intervention could improve female college students’ health behaviors, since previous studies have reported that eHL was a key component in promoting behavioral health outcomes [11,29]. However, the results of health behavior outcomes showed no significant interaction effects on exercise, eating, or sleep behaviors. In summary, eHL intervention was successful in increasing eHL among female college students, which is consistent with the previous intervention studies among adolescents [52] and older adults [12,42]. However, this did not lead to a significant increase in multiple positive health behaviors following the intervention. There might be a reason for the result that, after six weeks of intervention, the experimental group tended to improve overall health behavior, but the short intervention period may not have been sufficient to make healthy behavior a habit. This finding could be because intervention exposure duration is essential in changing college students’ health behavior as habits [21,53]. Therefore, in future studies, it is necessary to determine whether changes in health behavior outcomes occurred after a long-term period of intervention.

This study was conducted during COVID-19, when the stringent quarantine guidelines were preventing the rapid spread of COVID-19, causing physical inactivity [7,54], sleep problems [55], and eating problems [56] among college students. These might be another possible reason explaining the result that health behavior has not significantly improved after the intervention. This study’s PE control condition was a regular PE class during the COVID-19 period, and previous studies showed that health behaviors improved after participating in PE class [57,58,59]; however, our findings did not support this. Therefore, the COVID-19 factor and the short period of intervention exposure might have influenced its efficacy on health behavior change.

Whether longer exposure to the intervention resulted in a statistically significant health behavior change is unknown, but there was a significant improvement in cognitive exercise self-schemata after six weeks of exposure to the eHL education intervention. One of the key elements of the intervention content in this study was to acquire reliable health knowledge (e.g., exercise benefits and recommendations) through health education and related practice in order to increase motivation and interest in positive health behavior such as exercise. It supports the results that cognitive factors such as acquiring reliable health knowledge related to exercise can help form exercise self-schemata, a cognitive motivation to promote exercise participation [35,36]. While exercise self–schemata was improved, actual exercise behavior was not significantly improved, probably due to the intention and action gap [60]; the participants of the intervention did not report a significant increase in actual exercise behavior. Rhodes and de Bruijn conducted a meta-analysis to quantify the intention and behavior gap [60]. The results showed that 36% of the exercise intenders were not successful with their physical activity. Therefore, further research should continue to examine the effects of health education intervention to improve exercise motivation and involvement in physical activity. To prevent COVID-19, students were encouraged to stay at home, and all activities in the universities were changed to non-face-to-face, resulting in poor physical activity and a sedentary lifestyle [7]. This can be seen as the factor that reduced the students’ opportunity to participate in exercise, resulting in improved exercise self-schemata alone. In this study, we examined the effects of female students’ health behavior-related cognitive aspects by applying a real-life setting (e.g., PE class in college). Therefore, this study is differentiated from previous studies in that it is an evidence and practical study.

A notable result of this study was that after the intervention, the experimental group improved eHL, an essential factor in encouraging healthy lifestyles, and there was a slight improvement in health behaviors compared to the control group. Health behaviors such as exercise, specifically, were marginally significant at *p* < 0.10 [49]. It showed that the experimental group showed a marginally significant increase in exercise after the intervention, whereas the control group had no pre- and post-test difference. Given that eHL is becoming an increasingly important factor in public health outcomes [61], the findings contribute to the research literature through novel evidence that the intervention designed in this study can increase eHL and can positively affect health behaviors among female college students who are exposed to various health risks. Moreover, because college students’ health behaviors affect their future adulthood [1], providing eHL intervention to college students can help them make better health-related decisions based on trusted information on the Internet and maintain their healthy lifestyles throughout college and beyond. However, more studies are required to determine whether eHL intervention affects college students’ health behaviors and how long the intervention should last to promote positive health behaviors in college students.

This study has some limitations. First, we used purposive sampling from only female college students participating in PE classes, and data collection was conducted at the same college. Although our sample size (*n* = 120) is sufficient to provide appropriate statistical power, as in examples of other previous studies with similar numbers of female participants using randomized controlled trials [62,63], the results might be different if male students were included in this study as participants. Therefore, the findings may not represent the entire college-aged population, and hence, the results should be generalized cautiously. Therefore, further research is needed to confirm the validity of the intervention’s effectiveness using more diverse samples, such as medical students who require more eHL skills, using randomized controlled trials with sufficient power.

Second, the eHL intervention during PE classes used in this study was tested for its effectiveness compared with the control condition of participating in only PE classes. However, the intervention-integrated physical education and eHL education program are linked to each other, and hence, there is a limitation to identifying the individual effects of the eHL education program. Unlike the PE group, the intervention group showed a significant improvement in eHL, and hence, it was confirmed that the intervention is effective in enhancing eHL. Future studies with fully controlled groups are needed to examine the effectiveness of the two programs separately.

Finally, this study was a result of only one post-test measurement after a short intervention period, and the sustainability of the effect was not reviewed. Moreover, the outcome measures used were based on the self-reported questionnaire. Further studies are needed to add more objective or direct health-related outcome measures using mHealth devices (e.g., digital pedometer and a sleep pattern measurement app) to determine whether health behavior changes following the intervention would be observed and to check the continuity of the intervention effect through several follow-ups.

## 5. Conclusions

This study provides new evidence for the effects of online eHL intervention on improving eHL and promoting changes in the positive perception of exercise among female college students; although, multiple health-behavior changes were not apparent over the six-weeks intervention period. Consequently, online eHL education materials are well-designed and evaluated for female college students, given their vulnerability to health. The intervention might be a potential program for helping female students become more educated on eHL and might be sufficient as a means of improving multiple health behavior outcomes closely related to eHL. These preliminary findings have important implications for physical education teachers and researchers in health. There should be more focus on the intervention, including essential components related to improvements in eHL and positive health behaviors regarding their application in various health-related classes in the college education site, aiming for better healthcare and a healthier lifestyle. However, future research is needed to examine the potential effect of applying this type of intervention to diverse samples with low eHL (e.g., older adults, patients) over a longer period, thereby positively influencing multiple health behaviors by improving eHL.

## Figures and Tables

**Table 1:** eHealth literacy education program overview.

Session	Lesson Goal	Content
1	Knowledge-building #1: Learning positive health behavior and making it a habit: physical activity and health	Introduction to online eHealth literacy intervention and learning objectives Physical activity benefits and recommendations Checking current level of physical activity and setting physical activity plan
2	Knowledge-building #2: Learning positive health behavior and making it a habit: nutrition and healthy eating	Understanding basic healthy nutrition and proper eating habits for health Healthy dietary intake practices and recommendation Checking current level of nutrition intake and setting nutrition intake plan
3	Knowledge-building #3: Learning positive health behavior and making it a habit: sleep and health	Basic understanding and recommendation of healthy sleep Learning about factors that interfere with healthy sleep and how to eliminate them Checking current sleep habit and planning healthy sleep plan
4	Experience-building #4: Searching for health-related information using the Internet	Understanding the concept of eHealth literacy and its basic skills Checking out current eHealth literacy Searching for current health interests using the Internet Finding correct answers to health quizzes using the Internet
5	Experience-building #5: Knowing reliable health-related Internet websites	Introduction to reliable health information sites (e.g., Korea National Health Information Portal) and major literature search databases (e.g., PubMed, RISS) in medical and health sciences Retrieving all topics, A-Z pages, on the Korea National Health Information Portal website and finding health topics of one’s interest Finding journal articles and other types of materials on specific health topics on the Internet
6	Experience-building #6: Evaluating reliable health information and health websites	Learning how to distinguish the reliability of health information and websites on the Internet Critically evaluating health information and websites Review of all lessons and practices

**Table 2:** Participant characteristics and homogeneity test (*n* = 120).

Variables	EG (*n* = 62)	CG (*n* = 58)	χ^2^ or *t*	*p*
	*n* (%) or M ± SD	*n* (%) or M ± SD		
Age (years)	19.4 ± 0.5	19.5 ± 0.6	−1.34	0.182
Frequency of using Internet (hr./week)			2.82	0.831
<3	7 (11.3)	8 (13.8)		
≥3–<6	15 (24.3)	12 (20.7)		
≥6–<9	12 (19.4)	12 (20.7)		
≥9–<12	7 (11.3)	8 (13.8)		
≥12–<15	5 (8.0)	4 (6.9)		
≥15–<18	2 (3.2)	5 (8.6)		
≥18	14 (22.5)	9 (15.5)		
Subjective health status			5.89	0.117
Very healthy	2 (3.2)	0 (0)		
Healthy	18 (29.0)	22 (37.9)		
Moderate	29 (46.8)	31 (53.5)		
Unhealthy	13 (21.0)	5 (8.6)		
Health concern			3.97	0.265
Very interested	7 (11.3)	4 (6.9)		
Interested	19 (30.6)	22 (37.9)		
Moderate	24 (38.7)	27 (46.6)		
Little interested	12 (19.4)	5 (8.6)		
Exercise time (hr./week)			2.58	0.462
<1	36 (58.1)	31 (53.5)		
1–<3	17 (27.4)	22 (37.9)		
3–<6	8 (12.9)	5 (8.6)		
6–<9	1 (1.6)	0 (0)		
≥9	0 (0)	0 (0)		
eHL	3.36 (0.68)	3.53 (0.82)	−1.26	0.211
BESS	3.11 (0.82)	3.39 (0.88)	−1.82	0.071
CEESS	3.63 (0.68)	3.86 (0.77)	−1.76	0.080
Eating	2.67 (0.71)	2.69 (0.70)	−0.22	0.825
Sleep	2.91 (0.81)	3.03 (0.81)	−0.83	0.408
Exercise	3.23 (0.90)	3.07 (0.93)	0.96	0.341

Note: EG = experimental group; CG = control group; eHL = eHealth Literacy, BESS = behavioral exercise self-schemata; CEESS = cognitive-emotional exercise self-schemata; M = mean; SD = standard deviation.

**Table 3:** Changes in eHealth literacy over the intervention.

Variab.	Group	Pre-Test		Post-Test		G	T	G × T
		Mean (SD)	95% CI	Mean (SD)	95% CI	F(1, 118)	F(1, 118)	F(1, 118)
eHL	EG	3.36 (0.68)	3.17 to 3.55	3.82 (0.59)	3.66 to 3.98	0.064	8.277 **	4.765 *
	CG	3.53 (0.82)	3.34 to 3.73	3.59 (0.68)	3.43 to 3.76			

Note: Variab. = variables; SD = standard deviation; CI = confidence interval; eHL = eHealth literacy; EG = experimental group; CG = control group. * *p* < 0.05, ** *p* < 0.01.

**Table 4:** Changes in exercise self-schemata over the intervention.

Variab.	Group	Pre-Test		Post-Test		G	T	G × T
		Mean (SD)	95% CI	Mean (SD)	95% CI	F(1, 118)	F(1, 118)	F(1, 118)
BESS	EG	3.11 (0.82)	2.90 to 3.32	3.50 (0.63)	3.32 to 3.68	2.634	6.092 *	1.494
	CG	3.39 (0.88)	3.17 to 3.61	3.52 (0.77)	3.34 to 3.71			
CEESS	EG	3.63 (0.68)	3.45 to 3.81	3.92 (0.48)	3.77 to 4.07	0.026	0.611	5.648 *
	CG	3.86 (0.77)	3.67 to 4.05	3.71 (0.72)	3.55 to 3.87			

Note: Variab. = variables; SD = standard deviation; CI = confidence interval; BESS = behavioral exercise self-schemata; CEESS = cognitive-emotional exercise self-schemata; EG = experimental group; CG = control group. * *p* < 0.05.

**Table 5:** Changes in health behavior over the intervention.

Variab.	Group	Pre-Test		Post-Test		G	T	G × T
		Mean (SD)	95% CI	Mean (SD)	95% CI	F(1, 118)	F(1, 118)	F(1, 118)
Eating	EG	2.67 (0.71)	2.49 to 2.84	2.95 (0.75)	2.76 to 3.15	0.374	12.923 ***	0.142
	CG	2.69 (0.70)	2.51 to 2.88	3.05 (0.79)	2.85 to 3.25			
Sleep	EG	2.91 (0.81)	2.71 to 3.12	3.20 (0.82)	2.99 to 3.41	0.073	3.522	0.793
	CG	3.03 (0.81)	2.82 to 3.25	3.14 (0.87)	2.92 to 3.36			
Exercise	EG	3.23 (0.90)	3.00 to 3.47	3.52 (0.89)	3.29 to 3.75	10.793 **	0.245	3.474 ^†^
	CG	3.07 (0.93)	2.83 to 3.31	2.91 0.93)	2.67 to 3.15			

Note: Variab. = variables; SD = standard deviation; CI = confidence interval; EG = experimental group; CG = control group. ** *p* < 0.01, *** *p* < 0.001, ^†^ *p* < 0.10.

## Data Availability

The data included in the present study are available on request from the first author.

## References

[1] Daw J., Margolis R., Wright L. (2017). Emerging adulthood, emergent health lifestyles: Sociodemographic determinants of trajectories of smoking, binge drinking, obesity, and sedentary behavior. J. Health Soc. Behav..

[2] Nelson M.C., Story M., Larson N.I., Neumark-Sztainer D., Lytle L.A. (2008). Emerging adulthood and college-aged youth: An overlooked age for weight-related behavior change. Obesity.

[3] Davy S.R., Benes B.A., Driskell J.A. (2006). Sex differences in dieting trends, eating habits, and nutrition beliefs of a group of midwestern college students. J. Am. Diet. Assoc..

[4] Racette S.B., Deusinger S.S., Strube M.J., Highstein G.R., Deusinger R.H. (2005). Weight changes, exercise, and dietary patterns during freshman and sophomore years of college. J. Am. Coll. Health.

[5] Yahia N., Wang D., Rapley M., Dey R. (2016). Assessment of weight status, dietary habits and beliefs, physical activity, and nutritional knowledge among university students. Perspect. Public Health.

[6] Kecojevic A., Basch C.H., Sullivan M., Davi N.K. (2020). The impact of the COVID-19 epidemic on mental health of undergraduate students in New Jersey, cross-sectional study. PLoS ONE.

[7] Dziewior J. (2021). Examining Changes in Physical Activity and Sedentary Behavior of College Students During the Coronavirus Disease Pandemic (COVID-19). Master’s Thesis.

[8] Norman C.D., Skinner H.A. (2006). eHEALS: The eHealth literacy scale. J. Med. Internet Res..

[9] Smith A., Rainie L., Zickuhr K. College Students and Technology. https://policycommons.net/artifacts/624103/college-students-and-technology/1605386/.

[10] Stellefson M., Hanik B., Chaney B., Chaney D., Tennant B., Chavarria E.A. (2011). eHealth literacy among college students: A systematic review with implications for eHealth education. J. Med. Internet Res..

[11] Hsu W., Chiang C., Yang S. (2014). The effect of individual factors on health behaviors among college students: The mediating effects of eHealth literacy. J. Med. Internet Res..

[12] Watkins I., Xie B. (2014). Ehealth literacy interventions for older adults: A systematic review of the literature. J. Med. Internet Res..

[13] Ivanitskaya L., O’Boyle I., Casey A.M. (2006). Health information literacy and competencies of information age students: Results from the interactive online Research Readiness Self-Assessment (RRSA). J. Med. Internet Res..

[14] Dadaczynski K., Okan O., Messer M., Leung A.Y.M., Rosário R., Darlington E., Rathmann K. (2021). Digital health literacy and web-based information-seeking behaviors of university students in Germany during the COVID-19 pandemic: Cross-sectional survey study. J. Med. Internet Res..

[15] Escoffery C., Miner K.R., Adame D.D., Butler S., McCormick L., Mendell E. (2005). Internet use for health information among college students. J. Am. Coll. Health.

[16] Grim M., Hortz B., Petosa R. (2011). Impact evaluation of a pilot web-based intervention to increase physical activity. Am. J. Health Promot..

[17] Ince M.L. (2008). Use of a social cognitive theory-based physical-activity intervention on health-promoting behaviors of university students. Percept. Mot. Skills..

[18] Martens M.P., Buscemi J., Smith A.E., Murphy J.G. (2012). The short-term efficacy of a brief motivational intervention designed to increase physical activity among college students. J. Phys. Act. Health.

[19] Okazaki K., Okano S., Haga S., Seki A., Suzuki H., Takahashi K. (2014). One-year outcome of an interactive Internet-based physical activity intervention among university students. Int. J. Med. Inform..

[20] Rote A.E. (2017). Physical activity intervention using Fitbits in an introductory college health course. Health Educ. J..

[21] Duan Y.P., Wienert J., Hu C., Si G.Y., Lippke S. (2017). Web-based intervention for physical activity and fruit and vegetable intake among Chinese university students: A randomized controlled trial. J. Med. Internet Res..

[22] Plotnikoff R.C., Costigan S.A., Williams R.L., Hutchesson M.J., Kennedy S.G., Robards S.L., Allen J., Colins C.E., Callister R., Germov J. (2015). Effectiveness of interventions targeting physical activity, nutrition and healthy weight for university and college students: A systematic review and meta-analysis. Int. J. Behav. Nutr. Phys. Act..

[23] Zhang Y., Cooke R. (2012). Using a combined motivational and volitional intervention to promote exercise and healthy dietary behaviour among undergraduates. Diabetes Res. Clin. Pract..

[24] Harrer M., Adam S.H., Fleischmann R.J., Baumeister H., Auerbach R., Bruffaerts R., Cuijpers P., Kessler R.C., Berking M., Lehr D. (2018). Effectiveness of an Internet- and app-based intervention for college students with elevated stress: Randomized controlled trial. J. Med. Internet Res..

[25] Britt R.K., Collins W.B., Wilson K., Linnemeier G., Englebert A.M. (2017). Ehealth literacy and health behaviors affecting modern college students: A pilot study of issues identified by the American College Health Association. J. Med. Internet Res..

[26] Kim S.H., Son Y.J. (2017). Relationships between eHealth literacy and health behaviors in Korean adults. Comput. Inform. Nurs..

[27] Huang C.L., Yang S.C., Chiang C.H. (2020). The associations between individual factors, eHealth literacy, and health behaviors among college students. Int. J. Environ. Res. Public Health.

[28] Yang S.C., Luo Y.F., Chiang C.H. (2019). Electronic health literacy and dietary behaviors in Taiwanese college students: Cross-sectional study. J. Med. Internet Res..

[29] Tsukahara S., Yamaguchi S., Igarashi F., Uruma R., Ikuina N., Iwakura K., Koizumi K., Sato Y. (2020). Association of eHealth literacy with lifestyle behaviors in university students: Questionnaire-based cross-sectional study. J. Med. Internet Res..

[30] Yang S.C., Luo Y.F., Chiang C.H. (2017). The associations among individual factors, ehealth literacy, and health-promoting lifestyles among college students. J. Med. Internet Res..

[31] Roh M.Y. (2021). The effect of eHealth literacy on exercise self-schemata in female college students. J. Korean Phys. Educ. Sport Assoc. Girls Women.

[32] Kendzierski D. (1990). Exercise self-schemata: Cognitive and behavioral correlates. Health Psychol..

[33] Park I.K., Kim Y.H. (2013). College students’ stage of change, exercise self-schemata, and exercise adherence intention related to weekly exercise time. Korean J. Sport. Psychol..

[34] Yin Z., Boyd M.P. (2000). Behavioral and cognitive correlates of exercise self-schemata. J. Psychol..

[35] Berry T.R., Strachan S.M., Verkooijen K.T. (2014). The relationship between exercise schema and identity. Int. J. Sport Exerc. Psychol..

[36] Estabrooks P., Courneya K.S. (1997). Relationships among self-schema, intention, and exercise behavior. J. Sport Exerc. Psychol..

[37] Kim Y.B. (2010). Analysis of changes in health levels, health behaviors, and health-related factors among U.S. college students. J. Health Ed. Health Promo..

[38] Ministry of Culture, Sports and Tourism (2017). National Survey on Participation in Daily Exercise.

[39] Lee M.R. (2014). The effects of exercise self-schema and exercise self-efficacy on physical activity in the twenties. Korean J. Sports Sci..

[40] Choi S., Kim H. (2021). Current status and implications of health literacy among adults in Korea. Health Welf. Issues Focus..

[41] Lee M.R. (2021). The effect of online health promotion education programs on e-health literacy, affect, and wellness in pre-service childcare teachers. Korean J. Wellness.

[42] Xie B. (2011). Effects of an ehealth literacy intervention for older adults. J. Med. Internet Res..

[43] Won Y.K., Roh M.Y. (2021). The effect of health literacy education program on subjective health status, health interest and health literacy in female college students. Korean Women Phys. Educ..

[44] Glanz K., Rimer B.K., Viswanath K. (2015). Health Behavior: Theory, Research, and Practice.

[45] Ghaddar S.F., Valerio M.A., Garcia C.M., Hansen L. (2012). Adolescent health literacy: The importance of credible sources for online health information. J. Sch. Health.

[46] Lee B., Byun W., Lim J. (2010). The impact of an individual’s e-health literacy on physician-patient communication. J. Cyber Comm..

[47] Park I.K., Park S.H. (2012). Development and validation of measurement tools for exercise self-schemata. Korean J. Sport Psychol..

[48] Archibald M.M., Ambagtsheer R.C., Casey M.G., Lawless M. (2019). Using Zoom Videoconferencing for Qualitative Data Collection: Perceptions and Experiences of Researchers and Participants. Int. J. Qual. Meth..

[49] Carrera P., Caballero A., Muñoz D., González-Iraizoz M., Fernández I. (2014). Construal level as a moderator of the role of affective and cognitive attitudes in the prediction of health-risk behavioural intentions. Br. J. Soc. Psychol..

[50] Sheeran P., Orbell S. (2000). Self-schemas and the theory of planned behaviour. Eur. J. Soc. Psychol..

[51] Laar R.A., Ashraf M.A., Ning J., Ji P., Fang P., Yu T., Khan M.N. (2021). Performance, health, and psychological challenges faced by students of physical education in online learning during COVID-19 epidemic: A qualitative study in china. Healthcare.

[52] Paek H.J., Hove T. (2012). Social cognitive factors and perceived social influences that improve adolescent ehealth literacy. Health Commun..

[53] Epton T., Norman P., Dadzie A.S., Harris P.R., Webb T.L., Sheeran P., Julious S.A., Ciravegna F., Brennan A., Meier P.S. (2014). A theory-based online health behaviour intervention for new university students (U@uni): Results from a randomised controlled trial. BMC Public Health.

[54] Lin J., Guo T., Becker B., Yu Q., Chen S.T., Brendon S., Hossain M.M., Cunha P.M., Soares F.C., Veronese N. (2020). Depression is associated with moderate-intensity physical activity among college students during the COVID-19 pandemic: Differs by activity level, gender and gender role. Psychol. Res. Behav. Manag..

[55] Wang X., Chen H., Liu L., Liu Y., Zhang N., Sun Z., Lou Q., Ge W., Hu B., Li M. (2020). Anxiety and sleep problems of college students during the outbreak of COVID-19. Front. Psychiatry.

[56] Keel P.K., Gomez M.M., Harris L., Kennedy G.A., Ribeiro J., Joiner T.E. (2020). Gaining “the quarantine 15”: Perceived versus observed weight changes in college students in the wake of COVID-19. Int. J. Eat. Disord..

[57] Boyle J., Mattern C.O., Lassiter J.W., Ritzler J.A. (2011). Peer 2 peer: Efficacy of a course-based peer education intervention to increase physical activity among college students. J. Am. Coll. Health.

[58] Kimball J., Jenkins J., Wallhead T. (2009). Influence of high school physical education on university students’ physical activity. Eur. Phys. Educ. Rev..

[59] McKenzie T.L., Sallis J.F., Prochaska J.J., Conway T.L., Marshall S.J., Rosengard P. (2004). Evaluation of a two-year middle-school physical education intervention: M-SPAN. Med. Sci. Sports Exerc..

[60] Rhodes R.E., de Bruijn G. (2013). How big is the physical activity intention–behaviour gap? A meta-analysis using the action control framework. Br. J. Health Psychol..

[61] Chong Y.Y., Cheng H.Y., Chan H.Y.L., Chien W.T., Wong S.Y.S. (2020). COVID-19 pandemic, infodemic and the role of ehealth literacy. Int. J. Nurs. Stud..

[62] Eftekhari M.J., Peyman N., Doosti H. (2018). The effect of educational intervention based on the self-efficacy and health literacy theory on health promoting lifestyles among female health volunteers of Neyshabur, Iran. Health Dev. J..

[63] Kahtari M., Farmanbar R., Kasmaei P., Omidi S. (2017). The effect of the educational intervention on health literacy level in the girl students. J. Health Lit..

